# ‘A small cemetery’: death and dying in the contemporary British operating theatre

**DOI:** 10.1136/medhum-2019-011668

**Published:** 2019-07-25

**Authors:** Agnes Arnold-Forster

**Affiliations:** Humanities, University of Roehampton, London SW15 5PU, UK

**Keywords:** medical humanities, surgery, history, end of life care, doctor

## Abstract

Surgeon Henry Marsh begins his autobiography, *Do No Harm*, with a quotation from the French practitioner René Leriche, “Every surgeon carries within himself a small cemetery, where from time to time he goes to pray—a place of bitterness and regret, where he must look for an explanation for his failures”. This article uses memoirs and oral history interviews to enter the operating theatre and consider the contemporary history of surgeons’ embodied experiences of patient death. It will argue that these experiences take an under-appreciated emotional toll on surgeons, but also that they are deployed as a narrative device through which surgeons construct their professional identity. Crucially, however, there is as much forgetting as remembering in their accounts, and the ‘labour’ of death has been increasingly shifted out of the operating theatre, off the surgeons’ hands and into the laps of others. The emotional costs of surgical care remain understudied. Indeed, while many researchers agree that undergoing surgery can be a troubling emotional experience for the patient, less scholarly attention has been paid to the emotional demands performing surgery makes on surgical practitioners. Is detachment the modus operandi of the modern surgeon and if so, is it tenable in moments of emotional intensity—like patient death?

## Introduction

The neurosurgeon Henry Marsh begins his bestselling autobiography, *Do No Harm: Stories of Life, Death and Brain Surgery* (2014), with a quotation from the French surgeon René Leriche, “Every surgeon carries within himself a small cemetery, where from time to time he goes to pray—a place of bitterness and regret, where he must look for an explanation for his failures”.1 For a surgical memoir that explicitly attends to compassion and candour, this quotation is a fitting epigraph. It makes death manifest: a weight for the surgeon to shoulder, a map on which they can chart their personal and professional journey, and a delineated site within the surgical experience. Both Leriche and Marsh pay tribute to the emotional costs of surgery and centre death and regret in accounts of a profession for which routine care, digital interfaces and minimally invasive interventions are increasingly the norm.

The candid and compassionate surgical memoir, like *Do No Harm*, is a relatively new genre. Twenty years ago, you would be hard-pressed to find a single published autobiographical account by a surgeon. Those that you might uncover were narrative accounts of biographical detail, professional achievements and innovative successes.2 Instead, thick with emotional commentary, these recent surgical memoirs integrate accounts of professional and personal life and attest to the affective intensity of modern operative practice.3 They describe moments of doubt, failure and regret, and ruminate on the urgency and uncertainty of surgery. In the first few pages of *Do No Harm*, Marsh reflects on the tragic mysteries of complex operations,

I lay in bed thinking about the young woman I had operated on the previous week. She had a tumour in her spinal cord… and—although I do not know why, since the operation had seemed to proceed uneventfully—she awoke from the operation paralysed down the right side of her body… I longed for this next operation, the operation on the pineal tumour, to go well—for there to be a happy ending, for everybody to live happily ever after, so that I could feel at peace with myself once again.4

This passage reveals a professional reckoning with the emotional costs of care and the lasting affective impact unsuccessful surgery might have on its practitioners.

Memoirs in this genre try to construct the emotional landscape of contemporary surgery by emphasising the practitioners’ capacity for empathy and compassion. Indeed, *Do No Harm’s* blurb compels the reader to challenge their assumptions of neurosurgery as a ‘precise and exquisite craft, practised by calm and detached surgeons’.5 This notion of emotional ‘detachment’ is pervasive in literature and discourse about the role and identity of the doctor and the surgeon. Throughout the last century, commentators on the role and identity of the surgeon placed high value on detachment as they believed that emotions could interfere with a practitioner’s ability to effectively and efficiently carry out their work. Various observers and medical professionals argued that doctors must maintain ‘distance’ from their patients to, ‘generate objectivity in diagnosis and treatment’.6 This ‘detachment’ and maintenance of ‘distance’ requires surgeons to adopt and present an ‘aloofness’ from emotional investment in their patients.

This (un)emotional state has been transformed, however, into a caricature. There are persistent stereotypes of the surgeon that represent him as almost exclusively male, overconfident and unfeeling. This stereotype is prone to unpredictable outbursts of anger. He is volatile, insistent and even abusive. He cuts first, asks questions later—and he is never in doubt. He is good at hard surgeries but bad at ‘soft’ skills like compassion and communication. Academically brilliant, he lacks emotional intelligence. This trope is embodied by the fictional surgeon Sir Lancelot Spratt (played by James Robertson Justice), star of the 1954 film *Doctor in the House* (and its six sequels). Spratt, a detached, dispassionate demagogue, strode down hospital corridors with a team of frightened trainees hurrying along behind him.7 Sixty years on, this caricature still exists and continues to be influential on attitudes to surgery from within and without the profession. An article by Hill *et al*, published in 2014, explored medical students’ stereotypes of surgical careers and found that students described surgeons as self-confident and intimidating. Their perception that surgery is a competitive and masculine field, one that requires great personal sacrifice, deterred many from following a surgical career path. 8

This stereotype has also had lasting effects on the way we write about and research surgery. While policy-makers and medical educators have recently begun to investigate the potentially harmful effects of the vision of the surgeon as unemotional, unfeeling and antisocial in the uptake of surgical career paths and the gender disparities evident in the profession, few have taken a historical view of how this surgical caricature was shaped, maintained and on occasion undone.9 Moreover, the emotional costs of surgical care remain understudied. Indeed, while many researchers agree that undergoing surgery can be a troubling emotional experience for the *patient*, less scholarly attention has been paid to the emotional demands *performing* surgery makes on surgical *practitioners*.10 This blind spot is particularly true of the twentieth and twenty-first centuries as recent histories of Victorian medicine have problematised accounts of barbarous surgeons insensitive to the cries of un-anaesthetised patient suffering.11

However, historians have not paid similar levels of investigative attention to the place of emotions in twentieth-century and twenty-first-century surgery. We know little about what happened to surgical experience and identity and how those professional standards—dispassion, detachment and masculine stoicism—were challenged or maintained in the intervening century since the end of Queen Victoria’s reign. Is detachment still the *modus operandi* of the modern surgeon and if so, is it tenable in moments of emotional intensity—like patient death? To answer these questions—at least in part—this article uses memoirs and oral history interviews to enter the emotional landscape of the British hospital and consider the contemporary history of surgeons’ experiences of patients dying under the knife.

Much has changed over the course of the twentieth century and the surgeon–patient relationship has been profoundly altered by social, cultural and technological shifts. Surgery has become much safer and today your risk of an ‘intraoperative death’ is minimal, although not non-existent. While in 1900 you had a 50% chance of dying during or soon after an operation, today, you have a 3.6% chance of dying within 2 months of surgery and only between 1 and 30 in every 100 000 patients die on the table itself.12 As a result, some surgeons may go their entire career without ever witnessing such an event. A recent questionnaire survey in the *British Medical Journal* reported that only 53% of orthopaedic surgeons had ever experienced an intraoperative death.13 Of course, different subspecialties will be more or less likely to witness intraoperative death. Cardiac surgeons in their dealings with large blood vessels and brain surgeons in their involvement with the delicate structures inside the skull are more likely than their plastic or orthopaedic counterparts to preside over death on the operating table. It is, therefore, perhaps unsurprising that specialties with the potential for melodrama dominate the autobiography market—the recent surgical bestsellers have narrated the experiences of neurosurgery or cardiac surgery rather than the more quotidian stories of hip replacements and cataract removal. Moreover, the bestselling autobiographies have mostly been written by white men.14 Thus, while these memoirs provide rich source material for the subject of surgery and emotions, to diversify the gender, ethnicity and subspecialty of those experiences surveyed, I have conducted oral history interviews with 21 practitioners (6 women and 15 men), at multiple career stages and from a range of subspecialties, located all over the UK.15

Oral history is often conducted as a means of addressing topics and experiences that are missing from existing archives, and its practitioners have sought to record the experiences of the dispossessed, disempowered and marginalised.16 This valuable tendency has meant, however, that oral history interviews with doctors—who have substantial social and financial capital—are relatively rare.17 My corpus, therefore, constitutes one of the only existing collections of semistructured interviews with British surgeons.18 The oldest participant was born in 1938, and so the interviews cover surgical experiences from c.1950 to the present day. A semistructured interview is open, relatively freeform and allows new ideas to be brought up during the interview process.19 I began by asking participants about medical experiences in childhood, before moving chronologically through their lives according to a predetermined (although flexible and responsive) framework of themes. We discussed their decisions to embark on a medical career path, their early exposure to anatomical dissection, their first experiences on the hospital ward, their own experiences of ill-health, why they were drawn to surgery as a specialty, and their relationships with their colleagues, patients and families. The interviews each lasted for approximately an hour and were recorded and then transcribed. My theoretical approach to the analysis of the interviews is primarily historical and draws on the ample literature on reflexivity, trauma, and forgetting in oral history and autobiography to explore surgeons’ attitudes to, and narrations of, patient death.20 In particular, I deploy ideas about ‘forgetting’ and in line with historians such as Naomi Norquay suggest that like remembering, forgetting is an ‘active process’ that is key to identity-formation and the construction of coherent personal and professional narratives.21 Thus, I pay as much attention to the questions they cannot answer, as to those they can, and stay attuned to *how* they say something, not just *what* they say.

This article will draw on these surgeons’ responses to my questions to argue that patient death takes an under-appreciated emotional toll on surgeons, but also that they deploy it as a narrative device through which they construct their professional identity. Crucially, there is as much forgetting as remembering in their accounts, and the ‘labour’ of death has increasingly been shifted out of the operating theatre, off the surgeons’ hands, and onto intensive care physicians, palliative care professionals and nurses. Thus, death is a useful and provocative subject for discussion because it is emotionally intense and laden with cultural baggage, and also because surgical experiences of patient death have changed substantially over the last 60 years. I will begin by exploring some of the narratives surrounding death and dying that emerged from my interviews and are recounted in surgical memoirs, before analysing the shape of these narratives and asking why some deaths are remembered and others forgotten. Finally, this article will conclude with some reflections on the emotional landscape of British surgery and the systems, structures and support networks in place to alleviate feelings of grief, loss and trauma.

## My first death

I asked each surgeon to recall their first death because I found that it prompted participants to reflect on their earlier or earliest experiences of operative practice. For many, patient deaths that took place at the beginning of their careers were pivotal moments and prominent parts of their professional narrative. They often marked the commencement of their clinical identities—a transitional point through which everyone must pass to acquire their ‘medical selfhood’ and belong to a new community of practitioners.22 As anthropologists of medicine have acknowledged, the medical profession forces its participants to endure an intensive, rigorous and emotionally demanding apprenticeship.23 This is a process of socialisation that inculcates them into the values of the profession and cultivates the ‘medical identity’ that provides practitioners with status in both the medical and non-medical world. Patient deaths are a key component of that ‘apprenticeship’ and were frequently framed as opportunities for learning, whether that learning was part of a process of acclimatisation to the process of death and dying (what a dead body looks or feels like and sometimes framed as a process that cultivates ‘detachment’), or whether they provided more specific clinical lessons. The routine management of death and dying is, after all, one of the critical experiences that sets medically trained people apart from the rest of the population. It is also an experience that has remained relatively constant over the course of the NHS’ lifetime.

In the oral history interviews, I asked participants about their experiences of medical school and their encounters with death while still studying. Students’ first interaction with the dead is usually in the anatomy theatre where they first dissect bodies and parts of bodies. Almost all the surgeons reflected on that experience as being a positive one, something crucial to their intellectual development. One female consultant surgeon said, “We had a huge amount of anatomy… we had six to a dissecting table, we just did it ourselves, from top to bottom… I really really enjoyed that”

.24 She denied ever feeling ‘squeamish’ or unsettled and instead said, “I’ve always felt it’s an enormous privilege that we’re allowed to do that… we were very respectful”.25 That respect was partly due to constant reminders of the personhood and individuality of the body being dissected,

Now and again something would remind you. The weird thing is the hair. As the body shrinks the hair looks like its growing, like stubble, and that’s weird. Or there’ll be something that suddenly brings you back like a tattoo or a surgical scar or something like that and suddenly you’ll be jolted back to this was someone.26

She went on to say that despite these reminders, she was never ‘upset’ by the experience, “because you know that you’re doing it, you’re not mucking around, you’re learning how to do this for real”.27 She saw anatomical dissections as an essential part of clinical education and crucial for the development of surgical skill.

In the first few foundation years after completing their medical degrees, junior doctors are frequently called on to certify patient death and, for many, this process constitutes their first exposure to the dead and the materiality of their bodies. A female surgeon reflected on her experience of certifying deaths as a junior doctor in the early 1990s. In her interview, she described the intimacy of the process,

They’ve got the curtains round, and you get ushered in. And then they all disappear and leave you with your stethoscope, and you’ve got to spend a minute listening to the chest and watching this dead body. And you might never have seen a dead body, and this is your first time. You’re supposed to shine a light in their pupils, so you’ve got to look into their eyes… You’ve got to touch them and feel for a pulse… There’s a lot of time for reflection.28

In this narrative, the certification of patient death becomes a meditative process. Universally acknowledged signs of life (both clinical and cultural)—breath, eye contact, heartbeat—become signs of death that you must sit with, wait with, until you are sure. In his autobiography, Henry Marsh recalled a similar experience,

In my first year as a doctor… I would often be summoned, usually out of bed in the early hours, to certify the death of a patient… I would walk along the empty, anonymous corridors of the hospital, young and healthy and wearing a doctor’s white coat, to enter a dark ward and be directed by the nurses to a bed around which the curtains had been drawn.29

In both cases, the body was shrouded by drapes and secluded from the ward. There are various reasons why junior doctors are called on to do this job. It is low status and low stakes—there is little prestige associated with successfully certifying a dead body, and while mistakes might prove embarrassing, the potential for patient harm is minimal (although not without its costs to the dignity of the patient and their family). It offers, therefore, an opportunity for new doctors to familiarise themselves with death and dying. It prepares them (perhaps unintentionally) for those times when there is so much more at stake.

Common across both published memoirs and interviewed surgeons is the idea that patients are capable of accurately predicting their own deaths. When the surgeons I interviewed witnessed these prognostications early in their careers, these events influenced later perceptions of patient credibility. A female consultant surgeon remembered her experiences as a surgical house officer in 1991. She spoke about a patient who, “had an absolute premonition of doom and I didn’t know how to [um] alleviate that”.30 The patient died soon after predicting her own death, “she literally turned her face to the wall and died”.31 The surgeon reflected on how this experience affected her emotionally and influenced her later practice, “I could not shake her premonition… I completely believe patients when they have that feeling”.32 Henry Marsh made a similar observation, “It used to be called *angor animi*—the anguish of the soul—the feeling that some people have, when they are having a heart attack, that they are about to die. Even now, more than thirty years later, I can see very clearly the dying man’s despairing expression as he looked at me as I turned away”.33

Some have argued that the regularity of death and dying in the hospital—and the frequency with which surgeons are called on to commune with dead bodies—serves to detach practitioners from the emotional costs of surgical care. However, for many surgeons, emotional detachment is partial or incomplete, and some do not aspire to detachment as a professional standard at all. I interviewed a retired paediatric oncology surgeon who rejected the notion that practitioners should remain detached and emotionally distant when their patients died,

I don’t think parents [of his paediatric patients] regarded it as a negative when they saw that I got upset. I think some people think you’ve got to remain very detached and objective at all stages. I couldn’t do that, I was sometimes very involved with a particular child and the parents could see how upset I was. I don’t think any of those parents thought the less of me for it or thought it was a bad thing. But I do know doctors who think you shouldn’t really show your emotion. But I don’t agree with that really. I basically couldn’t hide it really, so I didn’t have a choice.34

In his book, *Admissions: A Life in Brain Surgery*, published in 2017 as a sequel to *Do No Harm*, Marsh reflects on the progressive disintegration of detachment as he aged and approached retirement, “But now that my surgical career was coming to an end, I could feel the defensive psychological armour that I had worn for so many years starting to fall away, leaving me as naked as my patients”.35

However, from *Do No Harm*, we can see that Marsh’s ‘psychological armour’, rather than once being intact, had always had its gaps. Specific conditions of patient death made it impossible for him to disregard the emotional consequences of surgical practice,

As a casualty officer, I would have to certify death in some poor soul who had collapsed and died in the street. On these occasions I would find their corpse fully dressed on a trolley, and having to undo their clothing to place my stethoscope on their heart was a profoundly different experience from certifying death in the hospital inpatients in their anonymous white gowns. I felt that I was assaulting them, and I wanted to apologise to them as I unbuttoned their clothes, even though they were dead. It is remarkable how much difference clothing makes.36

But it was not just clothes that could penetrate his self-protection, “I hated post-mortems and usually tried to avoid them. My detachment had its limits”.37

Detachment was particularly fragile in cases of patient death when surgeons said they felt responsible or if they doubted whether they had provided the best possible care. Many of Marsh’s early experiences of patient death stayed with him throughout his career, especially when his efforts had been in vain, “I have seen people die… I remember working all night trying, and failing, to save one man, fully awake and suffering horribly, who looked into my eyes as he bled to death from oesophageal varices”.38 Indeed, the affective demands placed by a death that is your responsibility as a doctor (even if the surgeon was not at fault in any legal or even medical capacity) are profound. This type of patient death can have complex and lasting emotional consequences on the presiding surgeons. About 25%–30% of the paediatric oncology surgeon’s patients died. When interviewed, he described the agonies of their parents, “The suffering they go through, is something that you just can’t help saying I hope this doesn’t happen to me”.39 Throughout the interview, he referenced his own children and his experiences as a parent, “I’ve got children and going back to the ‘70s, when my youngest children were small, I’d always come back from work and I would always go straight upstairs and go to their bedrooms and see if they were all right”.40 While all of these deaths had emotional consequences, he felt some more keenly than others. He recounted his feelings of guilt and regret, “There are patients I still have nightmares about because I feel I let them down and could have done better. Those will be with me until the day I die”.41

He was not alone in recalling some patient deaths with horror. When asked to describe a specific death, a female surgeon said, “I remember a dramatic one. There are a lot of deaths”. Her narrative was hurried and jumped back and forward in time, “I do remember a horrific death when I was a houseman of someone who we were planning to operate on. This looks really bad she’s all blue she looks terrible. Is she bad enough to call the cardiac arrest team? There was a prolonged arrest and she ended up in intensive care…” The consultant said, “We’ll have to do a pre-mortem”, and on the ward, while the patient was still alive, “he opened up her abdomen and put his hand in”. The woman died, and in her interview, the surgeon reflected on why this particular patient had stayed with her, “I just remember that as being really horrific. Because I’d known her and been talking to her and we’d been expecting to operate and she’d just gone blue at the edges and unresponsive. It was awful”.42 Often recalled with dread and regret, early experiences of patient death take their toll and discourses of emotional detachment barely feature in the interviews at all.

However, the interviewees’ willingness to discuss painful moments in their professional history with candour does not reflect surgeons’ general tendency to reflect emotionally on their practice. In a podcast made by the Royal College of Surgeons of England (RCS) on End of Life Care, consultant orthopaedic surgeon and former President of the RCS Dame Clare Marx suggested that, “as a profession we have been risk averse about having this conversation [about patient death]”.43 It is possible, therefore, that my interviewees do not reflect most current and recently retired surgical practitioners in their attitudes towards emotional detachment. Rather, they could instead represent a self-selecting group of surgeons attracted by my research, and by the opportunity to speak more frankly about their feelings. It is difficult to tell whether other surgeons, otherwise taciturn, would offer up similarly emotional reflections if given the time, space, and justification to do so.

While many participants recalled emotionally intense instances of patient death from early in their careers, many could not recall their ‘first deaths’ and some could not, or would not, reflect on patient death at all. One responded to my question with, “This is a long time ago. I don’t think I remember the first time”.44 However, while my prompts about death and dying were not always answered with accounts of loss, trauma and grief, there is little evidence to suggest that this ‘forgetting’ was the product of emotional detachment. For some, the realisation that they could not remember the first patient (or any subsequent ones) who died under their care was unnerving. They commented on how ‘terrible’ their forgetfulness was and fretted over what I (the interviewer) must think of them.45 While we might expect surgeons to recall early moments of emotional intensity vividly, it should come as no surprise that remembering and forgetting are not automatic processes. However, this leads us to the question: why are some moments of patient death remembered and others forgotten?

How and whether you remember an event depends on a range of variables, and not least your emotional state during the event itself. Memory is influenced by the actual events it records and also by other knowledge, experiences, expectations, interpretations, perceptions and feelings. When we store a memory, we not only record all sensory data—what we see, hear and smell, we also store our mood and emotional state. Emotionally intense experiences—like witnessing or being involved in a patient death—might be more likely to be remembered. And, of course, memories are not necessarily permanent. They can deteriorate over time, otherwise known as the concept of ‘transience’. Participants could struggle to remember such negative memories without the correct cues which may be absent from the setting and circumstances of an oral history interview. In what follows, I pose a few suggestions as to why some experiences of patient death might be remembered or forgotten, and reflect on the role played by these experiences in the construction of surgical identity.

It is worth pointing out that from my interviews there does not seem to be any identifiable change over time or any clear gendered or age-based responses. As many women as men ‘forgot’ their first deaths, and while we might expect older surgeons to be more reticent about emotions, I observed no such correlation. Rather, there seems to be a range of emotional landscapes in any given place and time. However, some surgeons reflected on the changing responsibilities and specialisation of practitioners and suggested that might have effected their experiences of patient death. I deal with this shift in the section on the ‘Restructuring of healthcare’.

### Senses of death

Patient death is a sensory experience as well as an emotional one. Those deaths that were remembered tended to be ones that had imprinted themselves on the bodies as well as the minds of the surgeons being interviewed. The senses have the capacity for bringing the past into the present at any time—unbidden, involuntarily—but they can also be deliberately foregrounded in the more formal setting of an oral history interview.46 Indeed, it is well known that senses can act as a mnemonic device or a memory trigger .47 Oral historians have used smells, tastes and materials to prompt participants to recall distant memories or to direct the conversation.48 Scholars such as Mark Paterson claim that, “it is through touch that we engage with the materiality of the world and it can invoke powerful memories involving other senses”.49 Precisely how the senses operate with memory is, so far, an imperfectly understood area by both neuroscientists and psychology. However, recognising the multisensory character of memory is crucial, “The unthinking dominance of the visual in accounts of the social limits our imagination and ignores the equally crucial role that other senses play in our experience and understanding of the world”.50

It is perhaps unsurprising that senses other than sight—and particularly touch—play a crucial role in the remembering of surgical events. Surgery is a manual profession, draws on haptic skill, and surgeons frequently express their identity in terms of their craft-like practice.51 When asked to recount their encounters with dead bodies and their experiences of patient death, descriptions of textures, smells and sounds dominate the interviews. When asked how she ‘felt’ about anatomical dissection, one female consultant surgeon immediately responded with thoughts on smell, “The first feeling is nausea, actually, because the smell of formalin is overwhelming and it sticks to clothes so you take it home with you”. She commented explicitly on the importance of smell in memory and recollection, “Recently I had the experience of going back to the fourteenth floor of the medical school for another reason… but you go out of the lift and that smell just took you back, right back to 1985”.

In 1996, a retired clinical physiologist and community physician recalled his first experience of the operating theatre as a 17-year-old son of a surgeon, working during the Second World War. Writing in the *British Medical Journal*, his description of this memory attests to the implication of senses and the emotions in remembering clinical experiences.52 The 17-year-old accompanied his father who had been called into the local children’s hospital to deal with two emergency night-time admissions. One patient, a 3-year-old unconscious girl with a crushed skull, was operated on in a crowded theatre. As his father withdrew the trephine, he had to staunch the bleeding with his left thumb. The surgeon called on his son to scrub in and help, “My finger had to replace his on the skull and I still recollect the sensation”. The girl died on the table. The article is entitled ‘Two Patients Who Determined My Career’, suggesting that this experience played a vital role in the development of his professional interests and identity.

In cardiac surgeon Stephen Westaby’s memoir, *Fragile Lives*, he begins by describing himself as a medical student watching an operation in Charing Cross hospital. The young woman on the table has a heart damaged by rheumatic fever. This was Westaby’s first death, and while he did not wield the scalpel himself, the experience clearly affected him. It opens his memoir and began his surgical life. He describes the materiality and tactility of the surgery in vivid detail,

Then, as if in slow motion, the cannula site in the aorta gave way. Whoosh! Like a geyser erupting, a crimson fountain hit the operating lights, spraying the surgeon and soaking the green drapes. Someone murmured, ‘Oh, shit.’ An understatement. The battle was lost. Before a finger could plug the hole the heart was empty. Blood dripped from the lights and red rivulets streamed across the marble floor. Rubber soles stuck to it. The anaesthetist frantically squeezed bags of blood into the veins, but to no avail. Life was fast ebbing away. As the injected slug of adrenaline wore off, the turgid heart simply blew up like a balloon and stopped. Stopped forever.53

The surgical registrar came to close over the wound, ready to move the body to the mortuary. Again, Westaby devotes paragraphs to describing the materiality of the body,

The young woman would be sliced open again from neck to pubis, so there was no point closing the breastbone or bringing together the different layers of the chest wall. He took a big needle and some thick braid, and sewed her up like a mailbag. The wound edges still gaped and oozed serum. Mail bags were much neater.54

He reflects explicitly on his capacity to remember this specific death.

Auxiliary nurses came with mops and buckets to erase the last traces of her—her blood now dry on the floor around the operating table, the bloody footprints heading towards the door, the blood on the anaesthetic machine, the blood on the operating lights. Blood everywhere—now meticulously wiped up… But one spot of blood remained on top of the light where no one could see. Adherent and black, it said part of me is still here. Remember me.55

There are many reasons why Westaby might have opened his autobiography with this story. It serves various narrative functions: it is dramatic, draws us into the memoir, and it evokes sympathy and compassion from the reader. It causes us to reflect on the rapid transformation of medical science and surgical efficacy even within one person’s lifetime (few Londoners will die from the complications of rheumatic fever today) and reveals the changing nature of surgery. It also shows how surgical death—and particularly cardiac surgical death—has the capacity to etch itself on the practitioners’ memory.

The woman’s visceral, viscous blood marked Westaby’s early experiences of surgery, and her mortal remains implored him to recall her, even 50 years later. The role played by senses in surgeons’ accounts of patient death also problematise the ideas about detachment set out in the introduction. Detachment is supposed to be a skill you cultivate—a process by which practitioners deliberately become capable of distancing themselves from the emotional intensity of patient care in order to be able to perform effectively and objectively. The cases outlined above suggest that detachment is unevenly applied and subject to the whims of circumstance and the variety of senses and emotions experienced in the operating theatre.

### Paradigm of surgery

Just as the notion of the ‘medical self’—or surgical identity—can explain why early experiences of patient death can prove pivotal, it might also explain why some surgeons do not remember these episodes. Halbwachs characterises memory as a filter that tends to preserve only those images that support the group or individual’s present sense of identity.56 Moreover, oral history can both destabilise and affirm a personal narrative.57 The stereotypical surgical identity is constructed from a group of traits that are traditionally considered masculine, such as ‘boldness of action’ and a ‘take-charge machismo’.58 For some, this might be about conforming to a detached or disinterested ideal. In these cases, recalling individual patients and their deaths might disrupt models of professionalism that privilege emotional distance. In a surgical textbook, published in 1965, the author wrote that the ‘typical surgeon’ must have “a rather more than normal degree of common sense which will enable him in his early stages of his career to inhibit his emotional responses to the sometimes tragic situations which confront him and get on with the job which needs to be done”.59

For others, patient death might be troubling (and therefore forgotten) because it disrupts what various practitioners interviewed called the ‘paradigm’ of surgery—the attribute that attracted them to the profession in the first place—that surgery, unlike medicine, is a practice of ‘doing’ and ‘fixing’.60 One surgeon put it thus, “The thing I loved about surgery, the thing I still love about surgery, is that people come to you with a problem, [er], which you diagnose and fix… That paradigm of sorting out problems, fixing them, moving on, I really responded to, I thought it was great”.61 In the RCS podcast on End of Life Care, Professor Keri Thomas, said that the ‘default position’ is to save life and that from a surgeon’s point of view “actually appreciating that people die goes against the grain”.62 Dame Clare Marx said, “as a group of people we are pro-active” and so coming to terms with patient death is particularly challenging to the surgical identity.63 Self-efficacy is a vital component of the mental training necessary for surgical performance and, therefore, death as the ultimate ‘failure’ of efficacy fits uneasily into any personal or professional narrative.

There is a pervasive perception that the surgeon’s (and, indeed, the doctor’s) role is to be responsible, to protect patients from themselves, and ultimately to cure disease and delay death. Uncertainty, error and doubt are, therefore, fundamentally problematic for a surgeon’s persona and sense of self: “a lot of doctors think that death is failure”.64 One surgeon said to me, “For me, I became a surgeon to fix people. Plus, I know, in myself, that I have only a finite amount of emotional energy to go around. So, when someone dies… I put them in a box, marked ‘I can’t fix you any more’. Then I take a deep breath and try and fix the things I can fix”.65 As oral historian Naomi Norquay argues, “what we do not remember, what we omit, and what we deem as not worth remembering is as important to identity construction as what we do remember, what we do include, and what is worth remembering”.66

### Restructuring of healthcare

In some ways, surgeons are the closest of all healthcare professionals to death. They hold their patients’ lives, quite literally, in their hands and this visceral connexion to death is less common among other doctors. Physicians are unlikely to have had an experience that can match, ‘for sheer horror’, their patient dying on an operating table. Surgeon Kevin J H Newman offered aortic aneurysm surgery as one of the more graphic examples: “alive one min, dead within seconds”.67 However, as I have established, dying on the operating table is increasingly rare. The same surgeon who commented on the ‘paradigm’ of surgery also said, “We’re lucky, we’re privileged in surgery, because if we can’t fix the problem, usually, someone else looks after them”, and in doing so identified another key reason why surgeons might be unwilling to discuss patient death. As the health service is restructured and increasingly specialised—and as surgery has become safer—surgeons encounter death less frequently. Thus, the ‘labour’ of death is increasingly shifted out of the operating theatre, off the surgeons’ hands and into the laps of others.68 These ‘others’ include intensive care physicians, nurses and palliative care professionals as well as the patients’ friends, families and carers. Kerri Thomas said that she did not “think it’s always at all the surgeon’s role to have an advanced care planning discussion”.69

Death in the operating theatre has become highly unlikely, and some subspecialties are very unlikely ever to preside over patient death. I asked an orthopaedic surgeon, “Do you feel you have much to do with death?” She responded, “Not generally any more. The good thing about orthopaedics is that we don’t generally make people ill—we don’t invade a body cavity. [Death] is an infrequent occurrence now—thank goodness”.70 Orthopaedic surgery is perhaps unusual in that respect, but across the surgical board, diseases that kill people are increasingly circumscribed by specialists, “There’s very little [bone] cancer work that’s done in a district general hospital. If we think something is malignant we refer it to a specialist centre”.71 This surgeon also no longer worked on acute trauma. Similarly, her colleagues who performed hip fracture repairs on elderly patients were then transferred to ortho-geriatricians who are experts in end-of-life care.

In this way, the emotional labour of dealing with death is transferred out of the operating theatre, off the shoulders of surgeons and onto other healthcare practitioners. We see this process taking place in Westaby’s account of the heart surgery at the beginning of his memoir,

Only the scrub nurse lingered. Then she was joined by the anaesthetic nurse who had comforted the patient in the anteroom. They took off their masks and stood silently for a while, unconcerned by the sticky blood that covered every surface and by the chest still splinted open. The anaesthetic nurse searched for the patient’s hand beneath the drapes and held it. The scrub nurse pulled away the blood-soaked covering from the face and stroked it.72

In this case, the surgeons leave the dead body behind, and only the nurses remain to perform any kind of emotional work in the aftermath of death. This differentiation of emotional labour is, of course, highly gendered. This operates along disciplinary lines with nurses—who tend to be female—taking on the care of the dying just as surgeons—who tend to be male—absent themselves from such efforts, engagements and relationships. The gendered dynamics of the emotional labour of death could also operate within the surgical profession—as female surgeons shoulder the bulk of the affective burden whereas male practitioners might find it easier to avoid. It is worth noting, however, that the male surgeons I interviewed were as likely, if not more likely, to reflect in emotional terms about their patients and professional experiences.

The decreasing likelihood that surgeons will preside over patient death is a product of many manifestly positive developments in healthcare. End-of-life care has become professionalised and there are now teams and individuals with specific expertise about caring for those approaching death. Moreover, and as shown above, surgery has become safer and intraoperative death increasingly rare. However, Dame Clare Marx suggested that this shift was not necessarily a good thing, “Most doctors don’t see a lot of death. Nurses see death. Carers see death… I’m not sure that a little more exposure to death and the different ways people die might be a good thing”.73 This suggestion can be interpreted as a recommendation for a subtle shift in the ‘surgical paradigm’ and a move away from what Professor Keri Thomas called the ‘default position’ to save life.74 Which is not to say that that should not be the primary goal of surgical intervention, but rather that there should be an acknowledgement of, and a more candid conversation about, the presence of death in twenty-first-century healthcare and the emotional costs it levies at practitioners.

## Emotional support

Despite the emotional intensity of the surgical experiences described in memoirs and interviews, only rarely did participants or authors comment on that emotional intensity being recognised by their colleagues and employers. The interviews reveal a lack of formal or systematic emotional or mental health support for surgeons, “There was never any formal support anywhere I worked for as surgeon”.75 Instead, practitioners filled that space with their own ad hoc and informal versions of comfort and care. For example, one paediatric surgeon offered his own form of bereavement counselling to the parents of his patients who had died and in doing so built and maintained a mutually beneficial relationship, “When a child has died I always offer the parents the opportunity to come back, as soon as they wanted, as often as they wanted. And some of them came back quite regularly just to talk it all through… I have parents who came back to see me years later, just to talk it all through”.76 Surgeons also often sought emotional support from friends and family, “The wife and family could sometimes see I was upset when I got home and if I wanted to talk to them about it I would”.77 Moreover, clinical systems could be repurposed for emotional support. Weekly morbidity and mortality meetings—designed to discuss clinical issues—“usually served as sort of a de-brief really”.78

However, various interviewees commented on how informal support networks within the surgical profession and the hospital had, over the past 30 years, collapsed. Nostalgia frequently inflected these conversations.79 When asked about the emotional costs of care, and their working conditions, and their experiences of patient death, almost all hark back to the halcyon days before the introduction of the European Working Time Directive (EWTD). The EWTD 48 hours working week entered law in European countries in 1998. A phased approach to implementation was agreed for doctors in training in the UK, which steadily reduced working hours to 58 in 2004, 56 in 2007 and 48 in 2009.80 A key component of the EWTD was that the maximum period of work for a resident without rest is 13 hours. Surgeons have argued that these shorter sessions of work have led to complex rotas and frequent handovers. The frequent handovers have made it difficult to maintain continuity of care with implications for patient safety and professional well-being alike.81

Indeed, the changing shape of surgical training and work was a thread running throughout all the interviews, intersecting with a range of issues, including patient death. Prior to the introduction of the EWTD and the MMC (Modernising Medical Careers)—surgeons trained as part of a ‘firm’—a hierarchical structure of senior and less-senior practitioners. Many of those interviewed attested to the supportive nature of this team approach—and argued that they could cope with 120 hours working weeks because they were maintained by a sense of comradery and mutual understanding. Many of the surgeons lamented the loss of the ‘family-like’ structure of the NHS in days-gone-by.

One surgeon interviewed, who was born in 1944 and first qualified in the early 1970s, told me about how 100 hours working weeks were normal. He reflected on how different the working culture of the NHS had become and remarked that he was “quite happy that [he] lived through that old era”.82 In this ‘old system’. he said that “you very much felt like you were part of a firm… Whatever you were doing… you were definitely working within the team and that gave a very strong feeling of belonging and commitment”.83 However, histories of surgery and hospital care in the 1960s, 1970s and 1980s reveal a very different vision of the emotional landscape of the NHS—a hierarchical, male-dominated and exhausting system that relied on nepotism and the wives who performed the household labour and childcare required to allow their consultant husbands to work uninterrupted.84 The ‘firm’ system—while lauded by many—was also critiqued by several surgeons I interviewed and particularly by female practitioners who argued that it was a macho culture with little time or space for emotional reflection, women or family life. It also reveals a recurring pattern of initiatives to try and improve the emotional resources available to practitioners. From the foundation of the NHS to the late 1970s, articles published in the medical press repeatedly call for the preservation of social spaces in the hospital dedicated to doctors, the protection of surgeons’ lunch hours, and the provision of psychoanalytic and therapeutic support for staff.85 These articles suggest that twentieth-century surgeons have not always been detached and past practitioners found value in affective as well as technical expertise.

Despite the nostalgia that obscures contemporary surgeons’ vision of the past, there is nonetheless a pervasive sense that practitioners today are profoundly dissatisfied with the emotional support available to them and particularly in instances of intraoperative death.86 Professor Sir Alfred Cuschieri argued that, “a death on the operating table of a patient is a harrowing experience for a surgeon. In my view, the surgeon is emotionally and mentally not in the frame of mind to continue to operate that day”.87 In response, Sheriff Albert Sheenan recommended that surgeons should not operate for 24 hours after the intraoperative death of an elective surgical patient.88 There are also various initiatives that endeavour to provide the therapeutic resources so many surgeons require. For example, Schwartz Rounds offer a space and time in a busy working day where all staff come together regularly to discuss the aspects of their jobs. Rounds follow a standard model and adhere to a pre-determined structure. They take place regularly—usually once a month for an hour at a time. A round can either be based on a single case, or explore a theme such as ‘when things go wrong’ or ‘when I made a difference’. A panel, composed of three staff, share their experiences for the first 15–20 min, and for the remainder of the hour, trained facilitators ask participants to share their thoughts and reflections on the stories and lead an open discussion. Rounds are confidential, in which patient and staff identities are protected, and can help staff feel more supported in their jobs by allowing them the time and space to reflect on their roles. I have attended several Rounds as an observer on the condition that I do not use the material in my research. Patient death is a frequent topic of conversation in Schwartz Rounds. The clinical authors in an article on the introduction of Rounds to the Royal Brompton and Harefield Hospital Trust reflected,

Many of us were brought up in an age when the metaphorical stiff upper lip still held sway. While we were allowed to have ‘feelings’ we were discouraged from ‘wearing them on our sleeve’. As busy practising physicians, anaesthetists and surgeons, we inevitably have taken on the treatment of extremely sick patients whose time in hospital, despite very high levels of effort, ended with the death of the patient… By taking time to debrief as a team and discuss what the experience of caring for a critically-ill patient was like, we can guard against the long-term effects of the stress of such challenging situations.89

Quantitative evidence shows that staff who attend rounds feel less stressed and isolated.90 For practitioners to maintain their own emotional health and provide compassionate care to their patients, staff must feel supported in their work and have outlets that allow them to deconstruct limiting notions of detachment and dispassion.

## Conclusion

This article has used memoirs, oral history interviews, and other sources to enter the emotional landscape of the British hospital and consider the contemporary history of surgeons’ experiences of patient death. I have argued that while the experience of patient death is central to the formation of surgical professional identity and medical selfhood, it takes an under-appreciated emotional toll on surgeons. Moreover, these moments of ill-managed emotional intensity coalesce into a broader feeling that practitioners are inadequately supplied with the time, space and support necessary to process the emotional costs of care in the modern NHS. And yet, my prompts about death and dying were not always answered with accounts of loss, trauma and grief. Instead, there was as much forgetting as remembering in the surgeons’ accounts, and I have tried to suggest some explanations for this apparent amnesia. As I have shown, however, there is little evidence to suggest that this ‘forgetting’ was the product of emotional detachment.

Indeed, this case study—patient death—problematises the surgical image and identity and complicates any assumption that surgeons are, or must be, detached or distant from their patients. Not only is detachment uneven, unstable and inapplicable to many of the cases surgeons encounter today, efforts to maintain detachment and accord to the stereotypes that dominate the profession have had profoundly negative consequences on surgeon well-being, professional diversity and patient care. Detachment is a damaging fiction that does harm to the various participants of twenty-first-century healthcare. There is no benefit, I would argue, to acquiring or maintaining the type of detachment that some surgeons claim to value.

Finally, while my project is a historical one, I am also interested in the future of surgery. As I have noted previously, death on the operating theatre has become highly unlikely—and that trend will probably continue. How, then, will the surgeons of the future cope when it does—inevitably—all go wrong—and they are faced with a dead or dying patient that they were not prepared for? How will surgeons, highly trained in shoulder replacements, gallbladder removal and hernia repair, deal with the fatal complications that have always been part of their profession?

## Notes

1. Henry Marsh, *Do No Harm: Stories of Life, Death and Brain Surgery*. London: Hachette, 2014.

2. Reginald Murray, *Surgical Roots and Branches*. London: Wiley Blackwell, 1990.

3. Paul Kalanithi, *When Breath Becomes Air*. London: Random House, 2016; Stephen Westaby, *Fragile Lives: A Heart Surgeon’s Stories of Life and Death on the Operating Table*. London: Harper Collins, 2017; Gabriel Weston, *Direct Red: A Surgeon’s Story*. London: Random House, 2009.

4. Henry Marsh, *Do No Harm*, 4.

5. Ibid.

6. Natalie J Lewis et al, “Emotional intelligence medical education: measuring the unmeasurable?”, *Adv Health Sci Educ* 10, no. 4 (2005): 339–55.

7. Ralph Thomas, *Doctor in the House,* (1954).

8. Elspeth J R Hill et al, “Can I cut it? Medical students’ perceptions of surgeons and surgical careers”, *Am J Surg* 208, no.5, (2014): 860–7.

9. Erin Dean and Erin, “The gender pay gap in surgery”, *The Bulletin of the Royal College of Surgeons* 99, no.1 (2017): 12–14; Kim Peters and Michelle Ryan, “Machismo in surgery is harming the specialty”, *BMJ* 348, (2014); Kevin J H Newman, “A surgeon’s view of excising the ‘surgeon ego’ to accelerate progress in the culture of surgery”, *BMJ* 364, no. 158 (2019).

10. Ellen M Redinbaugh et al, “Doctors’ emotional reactions to recent death of a patient: cross sectional study of hospital doctors”, *BMJ* 327, no. 7408, (2003): 185–9; Sofia C Zambrano et al, “How do surgeons experience and cope with the death and dying of their patients? A qualitative study in the context of life-limiting illnesses”, *World Journal of Surgery* 72, (2013): 935–44.

11. Michael Brown, “Surgery and emotion: the era before anaesthesia”, in Thomas Schlich (ed.), *The Palgrave Handbook of the History of Surgery,* (London: Palgrave Macmillan, 2017), 327–48.

12. Rupert M Pearse et al, “Mortality after surgery in Europe: a 7 day cohort study”, *The Lancet* 380 no.9847, (2012): 1059–65.

13. I C Smith and M W Jones, “Surgeon’s attitudes to intra-operative death: questionnaire survey”, *BMJ,* 322 (2001): 896–7.

14. Exceptions include Gabriel Weston, *Direct Red: A Surgeon’s Story,* London: Jonathan Cape, 2009; and Gautam Das, *Tender is the Scalpel’s Edge: Glimpses for the Casebook of a British Consultant Surgeon,* London: Notion Press, 2017.

15. In 2016, women made up 11.1% of consultant surgeons in England. https://www.rcseng.ac.uk/careers-in-surgery/women-in-surgery/statistics/ (Accessed 14 Aug 2018)

16. Lynn Abrams, *Oral History Theory,* London: Routledge, 2016, 96–7.

17. The exception is the British Library’s ‘Oral Histories of Medicine and Health Professionals’, https://www.bl.uk/collection-guides/oral-histories-of-medicine-and-health-professionals (Accessed 14 Aug 2018).

18. Ruth McDonald et al interviewed surgeons for their 2006 article: Ruth McDonald, Justin Waring and Stephen Harrison, ‘Modernization and nostalgia in a hospital operating theatre department’, *Sociology* 40, no. 6 (2006): 1097–115.

19. Valerie J Janesick, “Oral History Interviewing: Issues and Possibilities”, In Patricia Leavy (ed), *The Oxford Handbook of Qualitative Research*, Oxford: OUP, 2014.

20. Wendy Rickard, “Oral history ‘more dangerous than therapy’?: interviewees’ reflections on recording traumatic or taboo issues”, *Oral History* 26, no. 2 (1998): 34–48; Alan Wong, “Conversations for the real world: shared authority, self-reflexivity, and process in the oral history interview”, *Journal of Canadian Studies/Revue d’études canadiennes* 43, no. 1, (2009): 239–58.

21. N. Norquay, ‘Identity and forgetting’, *Oral History Review* 26, no.1, (1999): 1–21, 2

22. A recent article by Dr Clare Gerada on the question of surgical mental health, published in the *Bulletin of the Royal College of Surgeons*, argues that doctors have their own ‘unwritten group norms’, that are deeply engrained and have long histories. Clare Gerada, “Clinical depression: surgeons and mental illness”, *Bulletin of the Royal College of Surgeons* 88, no. 8, (2017): 260–3.

23. Simon Sinclair, *Making Doctors: An Institutional Apprenticeship*, London: Berg, 1997.

24. Interview with female surgeon born in 1967; interviewed by author, 24 Nov 2017.

25. Ibid.

26. Ibid.

27. Ibid.

28. Interview with female surgeon born in 1970; interviewed by author, 12 Jan 2018.

29. Henry Marsh, Do No Harm, 145.

30. Interview with female surgeon born in 1967; interviewed by author, 24 Nov 2017.

31. Ibid.

32. Ibid.

33. Henry Marsh, Do No Harm, 83.

34. Interview with male surgeon born in 1944; interviewed by author, 18 Jan 2018.

35. Henry Marsh, Do No Harm, 21.

36. Henry Marsh, Do No Harm, 146–7.

37. Ibid., 146.

38. Ibid.

39. Interview with male surgeon born in 1944; interviewed by author, 18 Jan 2018.

40. Ibid.

41. Ibid.

42. Interview with female surgeon born in 1970; interviewed by author, 12 Jan 2018.

43. Royal College of Surgeons, ‘End of Life Podcast Part One’, Podcast, 2018, https://soundcloud.com/user-385374793/eol-podcast-part-1

44. Interview with female surgeon born in 1979; interviewed by author, 23 Jan 2018.

45. Ibid.

46. Paula Hamilton, “The Proust Effect: Oral History and the Senses”, In Donald A. Ritchie (ed), *The Oxford Handbook of Oral History*. Oxford: OUP, 2011, 222

47. Geoffrey Cubitt, *History and Memory*. Manchester: Manchester University Press, 2007.

48. Hamilton, “The Proust Effect: Oral History and the Senses”, 220

49. Mark Paterson, *The Senses of Touch Haptics, Affects and Technologies*. Oxford: Berg, 2007, 8.

50. Hamilton, “The Proust Effect: Oral History and the Senses”, 220

51. Tiago Moreira writes about the ‘centrality of the body of the surgeon in surgery’. Tiago Moreira, “Coordination and embodiment in the operating room”, *Body & Society* 10 no.1 (2004): 109–29, 109. For an earlier example see, Thomas Schlich, “Why were surgical gloves not used earlier? History of medicine and alternative paths of innovation”, *Lancet*, (2015).

52. John Raison, “Two patients who determined my career: in at the deep end”, *BMJ* 312 no.7037, (1996), 1020.

53. Westaby, *Fragile Lives*, 5–6.

54. Ibid., 8.

55. Ibid., 11.

56. Quoted in Francesca Cappelletto, “Long-term memory of extreme events from autobiography to history”, *The Journal of the Royal Anthropological Institute* 9 no.2, (2003): 241–60, 241.

57. Wendy Rickard, “Oral history ‘more dangerous than therapy’?: interviewees’ reflections on recording traumatic or taboo issues”, 34–48.

58. Melanie Hammond Mobilio and Carol-Anne Moulton, “Surgeons’ Reactions to Error”, In Tobias S. Kohler and Bradley Schwartz, *Surgeons as Educators: A Guide for Academic Development and Teaching Excellence*. London: Springer, 2017: 316–17.

59. Hedley Atkins, *The Surgeon’s Craft*. Manchester: Manchester University Press, 1965, 30–1

60. J Cassell, “Of control, certitude and the ‘paranoia’ of surgeons”, *Culture, Medicine and Psychiatry* 11 no. 2, (1987): 229–49.

61. Interview with male surgeon born in 1966; interviewed by author, 18 Dec 2017.

62. Royal College of Surgeons. ‘End of Life Podcast Part One’. Podcast. 2018. https://soundcloud.com/user-385374793/eol-podcast-part-1

63. Ibid.

64. Ibid.

65. Interview with male surgeon born in 19; interviewed by author, 24 Nov 2017.

66. N. Norquay, ‘Identity and forgetting’, *Oral History Review* 26, no.1, (1999): 1–21, 2

67. Kevin J H Newman, “A surgeon’s view of excising the ‘surgeon ego’ to accelerate progress in the culture of surgery”, *BMJ* 364, no. 158 (2019).

68. Timothy G Buchman et al, “Who should manage the dying patient?: rescue, shame, and the surgical ICU dilemma”, *J Am Coll Surg* 194, no.5, (2002), 665–73

69. Royal College of Surgeons. 'End of Life Podcast Part One'. Podcast. 2018. https://soundcloud.com/user-385374793/eol-podcast-part-1

70. Interview with female surgeon born in 1967; interviewed by author, 24 Nov 2017.

71. Ibid.

72. Westaby, *Fragile Lives,* 6.

73. Royal College of Surgeons, ‘End of Life Podcast Part One’, Podcast, 2018, https://soundcloud.com/user-385374793/eol-podcast-part-1

74. Ibid.

75. Interview with male surgeon born in 1944; interviewed by author, 18 Jan 2018

76. Ibid.

77. Ibid.

78. Ibid.

79. Agnes Arnold-Forster, “Doctors’ wellbeing: learning from the past can help improve the future”, *BMJ,* (2018).

80. Richard Canter, “Impact of reduced working time on surgical training in the United Kingdom and Ireland”, *The Surgeon,* 9 (2011): 6–7.

81. Ibid.

82. Interview with male surgeon born in 1944; interviewed by author, 18 Jan 2018

83. Ibid.

84. Agnes Arnold-Forster, “Doctors’ wellbeing: learning from the past can help improve the future”.

85. J H Tweedie et al, “Facilities for junior doctors in new hospitals”, *BMJ* 2, no. 6150, (1978): 1503; J. F. Murray, “Improving the hospital service”, *BMJ* 1 no. 6008, (1976): 507–8; Anon, ‘The orthopaedic surgeon’, *BMJ* 1, no. 5698, (1970): 748–9; N. Davis and H. Stephen Pasmore, “Anxious patient and worried doctor”, *BMJ* 1 no. 5061, (1958): 40.

86. Marc R de Leval, “Facing up to surgical deaths: each death should be subjected to forensic and statistical analysis”, *BMJ* 328, no. 7436, (2004): 361–2.

87. M Jones, ‘Deaths on the operating table’, *BMJ* 320, no. 7238 (2000): 881.

88. I C Smith and M W Jones, ‘Surgeons’ attitudes to intraoperative death: questionnaire survey’, 896.

89. John R Pepper et al, ‘Schwartz Rounds: reviving compassion in modern healthcare’, *J R Soc Med* 105, no. 3, (2012): 94–5.

90. Jill Maben et al, ‘A realist informed mixed methods evaluation of Schwartz Center Rounds in England’, *Health Services and Delivery Research* 6, no. 37, (2018).
